# Dementia Family Caregivers’ Ambivalent Feelings and Cardiovascular Risk: Longitudinal Correlates

**DOI:** 10.1093/geroni/igab046.679

**Published:** 2021-12-17

**Authors:** Rosa Romero-Moreno, Carlos Vara-García, Samara Barrera-Caballero, Javier olazarán, Brent Mausbach, Roland von Känel, Ricardo Olmos, Andrés Losada-Baltar

**Affiliations:** 1 Universidad Rey Juan Carlos de Madrid, Madrid, Madrid, Spain; 2 Universidad Rey Juan Carlos, Madrid, Madrid, Spain; 3 Rey Juan Carlos University, Alcorcón, Madrid, Madrid, Spain; 4 HGU Gregorio Marañón,, Madrid, Madrid, Spain; 5 University of California San Diego, La Jolla, California, United States; 6 University Hospital Zurich, Zurich, Zurich, Switzerland; 7 Universidad Autónoma de Madrid, Madrid, Madrid, Spain

## Abstract

Cross-sectional data show that caregivers’ ambivalent feelings are associated with psychological distress. The association of ambivalent feelings with caregivers’ cardiovascular risk has not been studied. For this purpose we analyzed preliminary data from the Spanish Longitudinal Caregiving Spanish Longitudinal Study (CUIDA-LONG). One-year follow-up data were available for 96 dementia family caregivers. The following variables were assessed: sociodemographics, body mass index (BMI), disruptive behaviors, ambivalence, depressive symptomatology and cardiovascular risk with the inflammatory biomarker C-reactive protein (CRP). A hierarchical regression model was tested. Sociodemographic variables and change over time in stressors, ambivalence and depression were entered as predictors of change in CRP. 27% of the variance in CRP was explained through the model. More time since being a caregiver, higher BMI and greater increase in ambivalence contributed significantly to an increase in CRP. Ambivalent feelings contribute significantly to the cardiovascular risk of those who care for a relative with dementia.

